# Prediction without intervention: why fifteen years of no-show models have not closed the loop on patient adherence

**DOI:** 10.3389/fdgth.2026.1949255

**Published:** 2026-10-07

**Authors:** David Flagel

**Affiliations:** Doctor of Technology Program, Purdue University, West Lafayette, IN, United States

**Keywords:** appointment no-show, clinical decision support, digital health, learning health systems, machine learning, maternal health, patient adherence, predictive analytics

## Abstract

Fifty-two published studies since 2010 have modeled which patients will miss which appointments. Ambulatory no-show rates have moved only modestly. This Perspective asks why fifteen years of prediction research has produced so little change in care, and argues that the answer is architectural, not algorithmic. In most health systems, the no-show model, the patient outreach platform, and the clinical risk tool are separate tools run by separate teams. Prediction is now routine, and prediction-guided interventions have reduced no-shows in trials. None of the systems in the studies and reviews examined here sense the full behavioral signal of their own outreach, fuse behavioral prediction into clinical risk, or learn in routine practice from the interventions they deliver. I describe three failures behind this gap: outreach platforms that discard the behavioral data they generate, behavioral and clinical risk models that never exchange a feature, and interventions that are never measured. I then propose three design principles for a closed-loop alternative, and a research agenda to test them. Maternal health, where a missed prenatal visit can mean an unmonitored pregnancy complication, is the motivating case; the principles apply wherever outcomes depend on sustained engagement with care.

## 1 Introduction

Consider a patient at 28 weeks of pregnancy who misses a scheduled prenatal visit. Her chronic hypertension goes unmonitored for another month. The blood pressure trend that would have prompted intervention is never measured. By the time she presents, options have narrowed.

This scenario is neither rare nor unpredictable. Maternal mortality review committees in 36 states determined that more than 80% of pregnancy-related deaths in 2017–2019 were preventable (1). In every period the CDC's maternal mortality review committee dashboard reports, from 2017–2019 through 2022, the contributing factor classes documented most often in preventable pregnancy-related deaths include clinical skill and quality of care, knowledge, lack of continuity of care, and lack of access or financial resources (2); the last two surface in daily practice as appointments not kept. A state-level analysis attributed part of the 1997–2012 rise in maternal mortality (4.9%) to the proportion of births to women attending fewer than ten prenatal visits (3).

The research community's dominant answer to this problem has been to predict it. If we can identify, in advance, which patients will miss which appointments, the reasoning goes, providers can intervene. It is a sensible premise. What it has not produced, I will argue, is a system that intervenes.

## 2 The prediction plateau

No-show prediction may be the most replicated task in clinical machine learning. A 2025 review of 52 publications from 2010 to 2025 found reported accuracy anywhere from 52% to 99.44% (4), and the top of that range is not believable for a behavioral outcome. The methods vary, logistic regression through gradient boosting and neural networks, but the ingredients rarely do: scheduling data, demographics, clinical history. Neither do the findings. Prior no-shows predict future ones. Long lead times hurt (4, 5). Distance, age, and insurance type turn up again and again, and a few models add weather (6) or socioeconomic variables (4). Refinements keep arriving, interpretable models that explain individual predictions (6), cost-sensitive handling of class imbalance (7), and each one shows, at most, an incremental gain on retrospective data.

Then comes each study's closing paragraph. The model could inform targeted outreach. The findings might support future intervention studies (6). What the intervention would be is sometimes named, but who would deliver it and how anyone would know it worked sits outside the study, in every study. Prediction alone has not been shown to change attendance. Prediction coupled to a targeted intervention has. A rapid systematic review found seven randomized trials and one non-randomized study of model-selected interventions: targeted phone calls (three trials, median relative risk 0.61, moderate certainty), text messages (one trial of 158,669 appointments, 0.91, high certainty), patient navigation (one trial, 0.55), and overbooking in three studies with very low certainty of any effect (8).

Nor is prediction confined to research. One common application is overbooking, which treats the prediction as a capacity-management input and may harm the patient who does arrive (8, 9). Where a patient-directed response exists, it is not standardized (10), and even successful deployments may be unable to quantify their own outreach (11); published evaluations of routine deployments are typically uncontrolled before-and-after comparisons (9, 11); and no study has compared targeted with non-targeted reminders head-to-head, leaving the added value of targeting itself unclear (8). The behavioral signal that outreach generates is used in its thinnest form: whether the patient confirmed, which is among the strongest predictors in the models that include it (12). Learning from what interventions do has been demonstrated only in a 60-patient randomized trial (13), a non-profit maternal program (14), and a one-shot message experiment (15). Table 1 lays this out study by study; it was assembled from the three reviews cited (4, 8, 16) and a targeted search described in its note, is not a systematic review, and bounds the claims of absence in this article.

**Table 1:** No-show and adherence prediction studies, categorized by the stages of a closed loop.

Study (setting)	Sensing source	Prediction	Intervention	Workflow integration	Feedback/adaptation	Measured outcome (design)
Toffaha et al. (4) – review of 52 studies, 2010–2025	Scheduling, demographic, clinical data	Logistic regression most common (68%); gradient boosting, random forests, neural networks	Rarely evaluated; “limited intervention options” identified as a gap	Seldom described	None; “lack of feedback loops” identified as a gap	Discrimination metrics (AUC 0.75–0.95); attendance change not reported in most studies
Liu et al. (6) – pediatric hospital, United States	Scheduling, EHR, weather, maternal education	Interpretable deep learning; individual-level explanations	None (suggested as future work)	None	None	Discrimination (retrospective)
Reategui-Rivera et al. (7) – national telemedicine system, Peru (1.5M appointments)	Scheduling and clinical data	Multiple ML models with class-imbalance handling	None	None	None	Discrimination (retrospective)
Alananzeh et al. (12) – academic arthroplasty clinic, United States	EHR, scheduling data, and response to dispatched reminders (confirmation)	Calibrated machine-learning models (LASSO, random forest, XGBoost, CatBoost; AUC 0.80–0.81); confirmation and prior no-shows dominant features	None (targeted outreach proposed for prospective evaluation)	None	None	Discrimination and calibration (retrospective, *n* = 17,614); confirmation aOR 0.24
Gurol-Urganci et al. (17) – Cochrane review of SMS reminders	None (blanket reminders)	None; all patients reminded	SMS reminders vs. none	Automated messaging	None	Attendance improved vs. no reminders (RR 1.14, 95% CI 1.03–1.26; 7 RCTs, 5,841 participants; moderate-quality evidence)
Steiner et al. (18) – integrated health system, United States (two RCTs)	EHR and appointment history	Model developed from trial data (C-statistic 0.90) and used to risk-stratify results afterward, not to target the intervention	Single IVR or text reminder vs. none	Automated reminder system	None	Missed appointments 6.5% vs. 7.5% (RR 0.85) at the index clinic and 8.2% vs. 10.5% (RR 0.76) at the replication clinic (RCTs, *n* = 8,804 and 7,497); authors note highest-risk patients “might require more intensive intervention.”
Shah et al. (19) – academic primary care, United States (RCT)	EHR and appointment history	Model identifies high no-show-risk visits	Targeted reminder phone calls to predicted high-risk patients	Prediction directly routes intervention	None (one-shot)	No-show rate 22.8% vs. 29.2% (absolute risk difference –6.4%; RCT, *n* = 2,247 patients at >15% predicted risk)
Tarabichi et al. (20) – safety-net health system, United States (randomized QI study)	EHR	Random forest model (vendor-developed, locally trained; c-statistic 0.80), calibration checked in Black and white patient strata before deployment	Live scheduler reminder calls to patients at ≥15% predicted risk	Prediction routes calls within scheduling workflow	None (one-shot)	No-show rate 33% vs. 36% overall (*p* < 0.01); 36% vs. 42% among Black patients (*p* < 0.001), narrowing the disparity (randomized; *n* = 2,858 augmented care, 2,982 standard care)
Oikonomidi et al. (8) – rapid systematic review of predictive-model-based interventions	EHR and scheduling data (across included trials)	Varied models identifying high no-show risk	Model-targeted text reminders, phone calls, or patient navigators	Varied; trial-based, not routine deployment	None reported across included trials	7 RCTs + 1 non-RCT: targeted calls median RR 0.61 (3 RCTs, 4,959 appointments); targeted texts RR 0.91 (1 RCT, 158,669 appointments); navigators RR 0.55
Berliner Senderey et al. (15) – 14 hospitals, Israel	None	None	Nine SMS reminder variants (eight alternative messages vs. generic control), sent five days before the appointment	Automated messaging	Yes, for message variants (intervention-level learning)	No-show 14.2% vs. 21.1% for the best message (OR 0.69, 95% CI 0.67–0.76; randomized, *n* = 161,587); winner adopted system-wide, no further learning
AlSerkal et al. (11) – primary care network, United Arab Emirates	EHR and scheduling data	Random forest, 16 features (86% reported accuracy)	Coordinators contacted high-risk patients (call details unspecified)	Real-time operational dashboard	None described	No-show rate −50.7% (before-and-after, 135,393 appointments; no control arm; four of eight authors vendor-affiliated, no competing interests declared)
Murray et al. (9) – academic health system, United States (case study of a vendor EHR model)	EHR (vendor model; inputs included ethnicity, financial class, religion, BMI, prior no-shows)	Vendor no-show model as shipped in the EHR	Targeted outreach piloted; overbooking use rejected on equity grounds	Vendor model embedded in scheduling workflow	None	9% mean relative reduction in no-show rates vs. prior year, 12 clinics (uncontrolled)
Noori et al. (10) – large health system, United States (deployment logging)	EHR (vendor LightGBM model, 22 features, trained on >7M appointments)	Every appointment scored at scheduling and nightly for one week prior	Left to local practice; response “varies by department”	Scores surfaced in scheduling interfaces	None (logging only)	Deployed AUC 0.646 on normal days; performance degraded during severe weather (observational)
Klimis et al. (21) – SMS-based chronic disease programs, Australia	Message content and participant replies	ML classification of message intent and reply type; associations with engagement and stopping	None (retrospective analysis)	None	None	Engagement and premature stopping (retrospective)
Mate et al. (14) – maternal mHealth program, India (field study)	Listenership of automated voice messages	Restless multi-armed bandit predicts engagement drop	Health-worker service calls allocated under limited capacity	Weekly call-allocation policy	Yes; intervention effects learned offline from historical service-call data; weekly re-prioritization on observed engagement state	Engagement drops reduced ∼30% relative to control (three-arm field study, *n* = 23,003)
Lauffenburger et al. (13) – REINFORCE trial, diabetes, United States (RCT)	Electronic pill-bottle adherence data	Reinforcement learning estimates individual responsiveness to message content	Daily personalized text messages	Automated messaging adapted daily	Yes; daily adaptation on observed adherence	Absolute adherence +13.6% (95% CI 1.7–27.1) vs. no-message control (RCT, *n* = 60); the most complete loop, at small scale
Bashir et al. (16) – narrative review, adherence prediction in chronic disease	EHR, pharmacy, retrospective records	Varied ML approaches	Intervention pathways identified as largely absent	Workflow integration identified as unresolved	None	Retrospective discrimination; limited external validation; few prospective evaluations

Studies were identified from the three reviews cited in the text (4, 8, 16), their reference lists, and targeted searches of PubMed and Google Scholar conducted between July and September 2026 for no-show and adherence prediction studies reporting an intervention, a deployment, or a feedback mechanism; the search was not systematic, no screening counts were recorded, and the table should be read as the studies examined rather than as an exhaustive census. Rows are ordered from prediction-only studies, through reminder trials without prediction, prediction-targeted trials, routine deployments of prediction, and communication-sensing studies, to the closest approaches to a complete loop; review articles appear as synthesis rows. “None” indicates the stage was explicitly absent from the study design; “not reported” indicates the study did not evaluate it. Effect sizes are as reported by the cited studies and are not comparable across designs. The Noori et al. entry is a preprint.

aOR, adjusted odds ratio; AUC, area under the receiver operating characteristic curve; CI, confidence interval; EHR, electronic health record; OR, odds ratio; QI, quality improvement; RCT, randomized controlled trial; RMAB, restless multi-armed bandit; RR, relative risk.

The claim here is not that prediction has failed or been ignored. It is that the field has proven the value of connecting prediction to intervention, deployed prediction at scale mainly to manage capacity, sensed only the smallest slice of the signal its own outreach produces, and learned from its interventions only at small scale. No-show rates reported in prediction research commonly fall between 10% and 20% (4). Across four decades of studies worldwide, the average is about 23%, from 13% in Oceania to 43% in Africa, and the median reported rate has declined only a few points per decade (5). Whether discrimination has improved over that period is unknown; the review pools performance across years and does not track it (4). My argument does not depend on a plateau. A tenth gradient boosting model on scheduling data will not change attendance, because the field has been optimizing the wrong stage of the pipeline. Prediction was supposed to be an input. Somewhere along the way it became the product.

## 3 Why the loop stays open

If predicting and then intervening is a valid premise, why has the connection between the two stayed open for fifteen years? Three structural failures, and two conditions that keep them in place.

### 3.1 Communication platforms are built as delivery channels, not sensing instruments

Nearly every health system sends automated appointment reminders, and every reminder produces behavioral data: whether the patient responds, how fast, on which channel, and with what stated concern (“I do not have transportation,” “I can't miss work again”). Almost all of it is discarded once the reminder has done its administrative job. The one data stream that directly observes the patient's engagement with her care plan between clinical encounters is treated as plumbing rather than signal. Early machine-learning studies of program messages and participant replies have shown that message characteristics are associated with engagement and premature stopping, but that analysis remains retrospective and disconnected from the risk models that drive care (21).

### 3.2 Behavioral risk and clinical risk are modeled separately

There has been a lack of recognition that how well an individual adheres to their care plan is a strong predictor of future clinical risk: adherence to treatment regimens roughly triples the odds of a good outcome (22), and missed primary care appointments, adjusted for morbidity burden, carry a dose-related increase in all-cause mortality of up to threefold, and more than eightfold in patients with mental health conditions (23). If a pregnant woman stops attending her scheduled prenatal visits, that is not simply a scheduling problem. She is a higher clinical risk. However, in most implemented systems, these two types of models will never exchange features.

### 3.3 Interventions are fired and forgotten

A score indicating need may trigger an intervention, but few interventions are implemented as experiments with systematic variability; a recent comprehensive review of the no-show prediction literature names the absence of feedback loops from intervention outcomes as an unresolved gap (4), and the feedback column of Table 1 is empty for all but three studies (13–15). Whether an intervention worked for a given patient is rarely measured, so neither the model nor the intervention logic learns, and the system cannot tell which patients benefit from which intervention.

### 3.4 Why these silos persist

Two conditions sustain these silos. The first is a lack of trust in how models are developed and how they assign numerical risk to patients. Clinicians’ confidence in these predictions remains fragile without interpretable models based on recognizable clinical logic, and providers tend to reject scores they cannot explain (16). Information overload exacerbates this distrust; when decision support tools generate too many alerts perceived as irrelevant, clinicians start to dismiss them, regardless of their actual importance (24).

The second condition is regulatory, in two senses. Patient messages are identifiable data, and the laws that protect them keep those messages out of modeling environments in many organizations. More consequentially, fusing a behavioral score into a clinical risk model that drives escalation is the step that converts an operational scheduling tool into something likely to meet software-as-a-medical-device definitions under FDA clinical decision support guidance and the EU Medical Device Regulation, with the EU AI Act adding obligations for high-risk health systems; and the continuous adaptation the loop requires raises predetermined change control questions the field is still resolving. Closing the loop therefore requires privacy-preserving design, transparency, and a change-control plan as preconditions.

## 4 Three design principles for closing the loop

The remedy is not a better classifier. It is an architecture that connects sensing, prediction, intervention, and measurement into a single learning loop (Figure 1), defined by three design principles.

**Figure 1:**
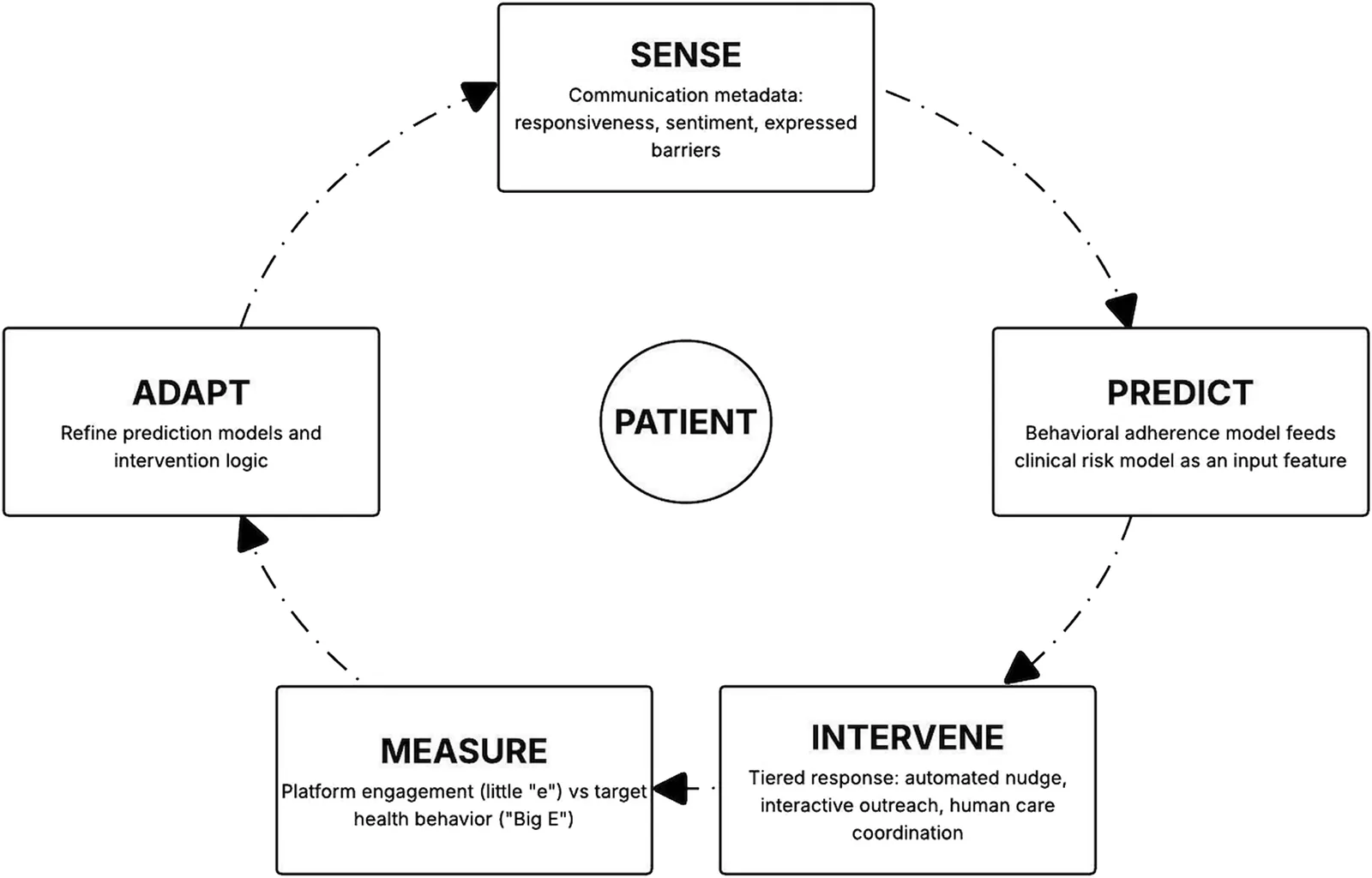
The closed-loop adherence architecture. SENSE: communication metadata is collected as an ongoing stream of behavioral features. PREDICT: a behavioral adherence model supplies input features to a clinical risk model. INTERVENE: elevated risk prompts a tiered response, from automated nudges to human care coordination. MEASURE: outcomes are evaluated on both platform engagement and the targeted health behavior. ADAPT: results feed back to both models and to the intervention logic.

### 4.1 Treat communication infrastructure as a sensing instrument

Every time a patient answers or ignores a reminder, the interaction leaves metadata behind, and those traces form a continually updating stream of behavioral features: response latency and channel choice index engagement; sentiment across messages tracks distress and fading motivation; natural language processing surfaces barriers that no claims feed contains. The case for sensing is not marginal discrimination; it is that a system which knows why a patient is at risk can match the intervention to the barrier. I call this stream “digital exhaust,” a byproduct of routine communication that becomes a real-time view of adherence between visits. The instrumentation exists in every deployed outreach system; what is missing is the decision to treat its output as clinical signal.

### 4.2 Fuse behavioral prediction into clinical risk

Because engagement behavior predicts clinical trajectory, behavioral adherence prediction belongs inside the clinical risk model as an input feature, not beside it. A model predicting a missed prenatal visit should do more than trigger a scheduling workflow; its score becomes a dynamic feature in the model stratifying that patient's risk of severe maternal morbidity, so the clinical score responds to the behavioral drift that so often precedes deterioration. There is precedent: adding socioeconomic status to Framingham coronary risk scoring did not change discrimination, but it corrected systematic underestimation of risk for low-SES adults and reclassified 15% of them to a higher treatment threshold (25).

Fusing the two models raises design questions any implementation has to answer. If the adherence score is recomputed as communication data arrive, the clinical score can move between visits, but every behavioral feature must then be built only from data that existed before the moment of prediction, or the clinical model ends up learning from signals that arrived after the outcome it was supposed to anticipate. The result looks excellent in retrospect and useless in deployment. Adding an input also unsettles a clinical model's calibration, and the adherence score is itself an estimate, and its error should propagate into the clinical prediction. Behavioral signals also drift quickly: a deployed no-show model lost discrimination during severe weather (10), and dataset shift in deployed clinical models is well described (26). The fused model needs drift monitoring on both inputs and a retraining cadence set by the faster of the two.

Fusion also creates a problem that drift monitoring does not solve. Once the adherence score both feeds the clinical model and triggers outreach, it stops being an observational predictor and becomes a quantity the system manipulates. The clinical model learned the relationship between adherence and outcome in a world where no one intervened on adherence; in the world the loop creates, high-risk patients are contacted and escalated, and the more effective the intervention, the weaker the association. A naive retraining cycle reads that attenuation as evidence that adherence no longer matters. The remedy is the experimental structure of Section 4.3: when delivery is randomized, assignment enters the fused model as a covariate and the adherence score's contribution to clinical risk stays identifiable. A loop that acts on its own predictions must randomize how it acts.

### 4.3 Treat every intervention as an experiment

What should a high-risk prediction trigger? A tiered response: automated nudges for simple cases, interactive outreach when the nudge fails, a human coordinator for the barriers automation cannot resolve. The point is that every intervention, at every tier, runs as an experiment, with variation built in and results measured.

Measured against what? “Little e” is platform engagement: the opens, replies, and clicks; “Big E” is the health behavior itself: an appointment kept or a prescription refilled (27). And a risk score answers who is likely to miss an appointment, not which intervention will change that: the first predicts outcome risk, the second predicts treatment effect, which varies across patients and can be learned only from variation in what they receive (28). Systematic variation, as in micro-randomized designs (29), generates the data from which intervention effects can be estimated for different kinds of patients; without it, the loop can identify risk but never learn what reduces it.

### 4.4 What this architecture adds to existing paradigms

The loop in Figure 1 should look familiar to readers of the learning health system literature. The Institute of Medicine defined a learning health system as one in which knowledge is captured as a by-product of care delivery and fed back into practice (30); the cycle proposed here is that idea applied to patient adherence. What the literature leaves unspecified is the mechanism: what to sense, how to combine signals, what to measure for the individual patient. The reminder trials in Table 1 produced a validated model and an effective intervention, tested separately, with no mechanism for linking them (18).

Three other paradigms each hold one piece. Just-in-time adaptive interventions have worked out when and how to intervene at the individual level, by sensing from the patient's own device (31), but have not taken on clinical risk. Clinical decision support puts a prediction in front of a clinician at the point of care (24), then stops. Digital phenotyping quantifies behavior moment by moment (32), but from the patient's phone rather than the health system's outreach platform. Set against adherence, these four are stages of one loop, and the two joints this article adds are missing from all of them: the outreach channel as a sensing instrument, and behavioral prediction as an input to clinical risk (Supplementary Table 1).

To be explicit about the standing of the claims in this article: it is established that adherence to treatment regimens predicts clinical outcomes and that missed appointments predict mortality (22, 23), that message characteristics are associated with engagement and disengagement (21), that adding a non-clinical input can correct a clinical risk score's calibration for a disadvantaged group (25), and that prediction-targeted interventions can reduce missed appointments in randomized trials (Table 1). This article proposes the connection. What remains to be validated is everything that follows from it: the fusion and sensing gains, transferability across institutions, and whether the closed loop prospectively improves attendance and outcomes (Section 5.2).

## 5 Discussion

### 5.1 Objections and hard problems

#### 5.1.1 Surveillance and consent

A system that analyzes the content and metadata of patient messages to predict medical need is a different thing from one that analyzes claims. Patients message their care team in confidence, not to contribute training data, and the adherence-prediction literature recognizes privacy and consent as acute ethical tensions (16). A closed loop therefore needs an explicit consent architecture: purpose limitation and data minimization, tiered consent that lets a patient accept reminders without accepting analysis, an opt-out that does not diminish care, audit logging of secondary use, and structural separation of the messaging environment from the modeling environment.

#### 5.1.2 Equity and the silence problem

Patients without a stable phone, a data plan, stable housing, or English produce little digital exhaust, so a system built on the digitally active knows least about its highest-barrier patients (16). Silence is a signal in its own right, not an empty field: data missing through digital exclusion are missing not at random, and a model trained only on the digitally active will misestimate risk for the rest. The risk is manageable by design: a model checked for calibration in Black and white patient strata before deployment drove live reminder calls in a safety-net system, reducing no-shows predominantly among Black patients and narrowing the disparity (20). Without that discipline, the closed loop amplifies existing inequities with algorithmic confidence (16, 33).

#### 5.1.3 The evidence bar

A loop that converts every elevated score into a coordinator task only moves the bottleneck and reproduces the alert fatigue that already undermines clinical decision support (24); the tiers are load-bearing, reserving human attention for the cases that need it, with the contributing factors attached. Retrospective discrimination, the currency of the prediction literature, will never be sufficient for a system that acts on its own predictions, in a literature where even external validation remains limited (16). Closed-loop systems must be evaluated prospectively, yet a platform that continually reallocates interventions resists a fixed-protocol randomized trial. Micro-randomized trials and the methodology built for just-in-time adaptive interventions are the more promising route (29, 31), and the evaluation framework is itself an open question.

### 5.2 A research agenda

The architecture breaks down into questions the field can answer now. Fusion: how much does clinical risk prediction improve when a behavioral adherence score is added as an input, and does the gain persist once outreach is delivered on the score? The first half is a retrospective comparison on linked data; the second requires randomized delivery. Sensing: do communication-derived features identify actionable barriers that EHR, claims, and social determinants features cannot, and does matching interventions to those barriers change attendance? Generalizability: do privacy-preserving approaches such as federated learning produce adherence models that hold up at unseen institutions, without which none of this leaves the academic centers where models are built?

A pathway can be stated now, in four phases. First, the retrospective comparisons above, which require only linked data. Second, silent deployment, in which the fused model scores patients prospectively but triggers no action. Third, micro-randomized trials that randomize intervention components at each decision point, which estimate the effect of each tier for each kind of patient and preserve the identifiability of the fused model (29). Fourth, a stepped-wedge rollout across clinics, which measures the effect of the whole loop while every clinic eventually receives it (34).

Return, finally, to the patient at 28 weeks. In the system the field has spent fifteen years building, her missed appointment becomes one more row in next year's training set. In the system it should be building, the unanswered messages three weeks earlier had already moved her adherence score; the transportation barrier she mentioned in passing had already been flagged; the ride had already been offered; and if she still did not arrive, her care team knew the same day, and knew why. The difference between those two systems is not a better algorithm. It is the decision to close the loop.

## Data Availability

The original contributions presented in the study are included in the article/Supplementary Material, further inquiries can be directed to the corresponding author.

## Supplementary material

Supplementary Table 1 The proposed closed-loop adherence architecture compared with four established paradigms.

## References

[1] Trost S Beauregard J Chandra G Njie F Berry J Harvey A. Pregnancy-Related Deaths: Data from Maternal Mortality Review Committees in 36 US States, 2017–2019. Atlanta, GA: Centers for Disease Control and Prevention (2022).

[2] Centers for Disease Control and Prevention. Pregnancy-Related Deaths: Data from Maternal Mortality Review Committees [dashboard; Preventability and Contributing Factor Class tab; year selections 2017–2019, 2020, 2021, and 2022; page updated April 20, 2026]. Maternal Mortality Prevention (2026). Available online at: https://www.cdc.gov/maternal-mortality/php/data-research/mmrc/index.html (Accessed September 19, 2026)

[3] Nelson DB Moniz MH Davis MM. Population-level factors associated with maternal mortality in the United States, 1997–2012. BMC Public Health. (2018) 18:1007. 10.1186/s12889-018-5935-2 30103716 PMC6090644

[4] Toffaha KM Simsekler MCE Omar MA ElKebbi I. Predicting patient no-shows using machine learning: a comprehensive review and future research agenda. Intell Based Med. (2025) 11:100229. 10.1016/j.ibmed.2025.100229

[5] Dantas LF Fleck JL Cyrino Oliveira FL Hamacher S. No-shows in appointment scheduling—a systematic literature review. Health Policy. (2018) 122:412–21. 10.1016/j.healthpol.2018.02.002 29482948

[6] Liu D Shin W-Y Sprecher E Conroy K Santiago O Wachtel G. Machine learning approaches to predicting no-shows in pediatric medical appointment. npj Digit Med. (2022) 5:50. 10.1038/s41746-022-00594-w 35444260 PMC9021231

[7] Reategui-Rivera CM Cui W Escobar-Agreda S Rojas-Mezarina L Finkelstein J. Machine learning-based prediction of no-show telemedicine encounters. Telemed Rep. (2025) 6:109–19. 10.1089/tmr.2025.0009 40630810 PMC12235123

[8] Oikonomidi T Norman G McGarrigle L Stokes J Van Der Veer SN Dowding D. Predictive model-based interventions to reduce outpatient no-shows: a rapid systematic review. J Am Med Inform Assoc. (2023) 30:559–69. 10.1093/jamia/ocac242 36508503 PMC9933067

[9] Murray SG Wachter RM Cucina RJ. Discrimination by artificial intelligence in a commercial electronic health record: a case study. Health Affairs Forefront (2020). 10.1377/forefront.20200128.626576

[10] Noori A Boussina AE Bich HH Anibal J Maslinski J Burger M. A global log for medical AI. arXiv [Preprint] arXiv:2510.04033v2 (2026). 10.48550/arXiv.2510.04033

[11] AlSerkal YM Ibrahim NM Alsereidi AS Ibrahim M Kurakula S Naqvi SA. Real-time analytics and AI for managing no-show appointments in primary health care in the United Arab Emirates: before-and-after study. JMIR Form Res. (2025) 9:e64936. 10.2196/64936 39763194 PMC11729783

[12] Alananzeh R Khabaz K Liu T Newman-Hung NJ Stavrakis AI Wessel LE. Behavioral and engagement predictors of arthroplasty clinic no-shows: a calibrated machine learning analysis. Arthroplasty Today. (2026) 40:102096. 10.1016/j.artd.2026.102096 42598448 PMC13471054

[13] Lauffenburger JC Yom-Tov E Keller PA McDonnell ME Crum KL Bhatkhande G. The impact of using reinforcement learning to personalize communication on medication adherence: findings from the REINFORCE trial. npj Digit Med. (2024) 7:39. 10.1038/s41746-024-01028-5 38374424 PMC10876539

[14] wMate A Madaan L Taneja A Madhiwalla N Verma S Singh G. Field study in deploying restless multi-armed bandits: assisting non-profits in improving maternal and child health. In: AAAI (2022), 36, 12017–25. 10.1609/aaai.v36i11.21460

[15] Berliner Senderey A Kornitzer T Lawrence G Zysman H Hallak Y Ariely D. It’s how you say it: systematic A/B testing of digital messaging cut hospital no-show rates. PLoS One. (2020) 15:e0234817. 10.1371/journal.pone.0234817 32574181 PMC7310733

[16] Bashir SG Salad HA Abdullahi YB Abdi YH Abdi MS Ahmed NI. Artificial intelligence approaches to predicting treatment non-adherence in chronic diseases: a narrative review. Front Digit Health. (2026) 8:1769337. 10.3389/fdgth.2026.1769337 42058725 PMC13121239

[17] Gurol-Urganci I De Jongh T Vodopivec-Jamsek V Atun R Car J. Mobile phone messaging reminders for attendance at healthcare appointments. Cochrane Database Syst Rev. (2013) 2013:CD007458. 10.1002/14651858.CD007458.pub3 24310741 PMC6485985

[18] Steiner JF Shainline MR Bishop MC Xu S. Reducing missed primary care appointments in a learning health system: two randomized trials and validation of a predictive model. Med Care. (2016) 54:689–96. 10.1097/MLR.0000000000000543 27077277

[19] Shah SJ Cronin P Hong CS Hwang AS Ashburner JM Bearnot BI. Targeted reminder phone calls to patients at high risk of no-show for primary care appointment: a randomized trial. J Gen Intern Med. (2016) 31:1460–6. 10.1007/s11606-016-3813-0 27503436 PMC5130951

[20] Tarabichi Y Higginbotham J Riley N Kaelber DC Watts B. Reducing disparities in no show rates using predictive model-driven live appointment reminders for at-risk patients: a randomized controlled quality improvement initiative. J Gen Intern Med. (2023) 38:2921–7. 10.1007/s11606-023-08209-0 37126125 PMC10150669

[21] Klimis H Nothman J Lu D Sun C Cheung NW Redfern J. Text message analysis using machine learning to assess predictors of engagement with mobile health chronic disease prevention programs: content analysis. JMIR Mhealth Uhealth. (2021) 9:e27779. 10.2196/27779 34757324 PMC8663456

[22] DiMatteo MR Giordani PJ Lepper HS Croghan TW. Patient adherence and medical treatment outcomes: a meta-analysis. Med Care. (2002) 40:794–811. 10.1097/00005650-200209000-00009 12218770

[23] McQueenie R Ellis DA McConnachie A Wilson P Williamson AE. Morbidity, mortality and missed appointments in healthcare: a national retrospective data linkage study. BMC Med. (2019) 17:2. 10.1186/s12916-018-1234-0 30630493 PMC6329132

[24] Sutton RT Pincock D Baumgart DC Sadowski DC Fedorak RN Kroeker KI. An overview of clinical decision support systems: benefits, risks, and strategies for success. npj Digit Med. (2020) 3:17. 10.1038/s41746-020-0221-y 32047862 PMC7005290

[25] Fiscella K Tancredi D Franks P. Adding socioeconomic status to Framingham scoring to reduce disparities in coronary risk assessment. Am Heart J. (2009) 157:988–94. 10.1016/j.ahj.2009.03.019 19464408

[26] Finlayson SG Subbaswamy A Singh K Bowers J Kupke A Zittrain J. The clinician and dataset shift in artificial intelligence. N Engl J Med. (2021) 385:283–6. 10.1056/NEJMc2104626 34260843 PMC8665481

[27] Cole-Lewis H Ezeanochie N Turgiss J. Understanding health behavior technology engagement: pathway to measuring digital behavior change interventions. JMIR Form Res. (2019) 3:e14052. 10.2196/14052 31603427 PMC6813486

[28] Kent DM Paulus JK Van Klaveren D D'Agostino R Goodman S Hayward R. The predictive approaches to treatment effect heterogeneity (PATH) statement. Ann Intern Med. (2020) 172:35–45. 10.7326/M18-3667 31711134 PMC7531587

[29] Klasnja P Hekler EB Shiffman S Boruvka A Almirall D Tewari A. Microrandomized trials: an experimental design for developing just-in-time adaptive interventions. Health Psychol. (2015) 34S:1220–8. 10.1037/hea0000305 26651463 PMC4732571

[30] Institute of Medicine. Best Care at Lower Cost: The Path to Continuously Learning Health Care in America. Washington, D.C.: National Academies Press (2013). 10.17226/13444 24901184

[31] Nahum-Shani I Smith SN Spring BJ Collins LM Witkiewitz K Tewari A. Just-in-time adaptive interventions (JITAIs) in mobile health: key components and design principles for ongoing health behavior support. Ann Behav Med. (2018) 52:446–62. 10.1007/s12160-016-9830-8 27663578 PMC5364076

[32] Torous J Kiang MV Lorme J Onnela J-P. New tools for new research in psychiatry: a scalable and customizable platform to empower data driven smartphone research. JMIR Ment Health. (2016) 3:e16. 10.2196/mental.5165 27150677 PMC4873624

[33] Jean-Francois B Bailey Lash T Dagher RK Green Parker MC Han SB Lewis Johnson T. The potential for health information technology tools to reduce racial disparities in maternal morbidity and mortality. J Womens Health (Larchmt). (2021) 30:274–9. 10.1089/jwh.2020.8889 33211604 PMC8020554

[34] Hemming K Haines TP Chilton PJ Girling AJ Lilford RJ. The stepped wedge cluster randomised trial: rationale, design, analysis, and reporting. Br Med J. (2015) 350:h391. 10.1136/bmj.h391 25662947

