# Supplementary material for: Prediction without intervention: why fifteen years of no-show models have not closed the loop on patient adherence
Source: Front Digit Health. 2026 Oct 7;8:1949255. doi: 10.3389/fdgth.2026.1949255 (PMC13683762; doi:10.3389/fdgth.2026.1949255)
Supplement: Supplementary file 1 [file Table1.pdf]

Supplementary Material

Supplementary Table 1 The proposed closed-loop adherence architecture compared with four established paradigms.

Paradigm	Level of operation	Sensing source	Prediction target	Intervention locus	Learning loop	Left unspecified for adherence
**Learning health system (30)**	Institution / population	Routine care data (EHR, claims, quality data)	Population-level practice questions	Practice change via quality improvement and guidelines	Yes, at institutional cadence (months to years)	What to sense, how to fuse behavioral and clinical signals, what to measure at the individual level
**Just-in-time adaptive intervention (31)**	Individual	Personal devices, self-report, wearables	Momentary receptivity and need for support	Personal-device prompts and messages	Yes, at individual cadence (hours to days)	Clinical risk; institutional workflow; population reach beyond device owners
**Clinical decision support (24)**	Clinical encounter	EHR	Clinical risk or guideline deviation	Alerts and recommendations to clinicians	No; rules or trained models fixed between updates	Patient-facing intervention; measurement of intervention effect; adaptation
**Digital phenotyping (32)**	Individual	Smartphones and other personal digital devices	Behavioral and mental-health phenotypes	None intrinsic (measurement paradigm)	No	Intervention, workflow, and consent context of institutional data; reach limited to device owners
**Proposed closed-loop adherence architecture (this Perspective)**	Individual patient within institutional operations	Institutional communication infrastructure (digital exhaust) plus EHR	Adherence behavior, fused as an input to clinical risk	Tiered outreach instrumented as experiments	Yes, at intervention cadence, measured against platform engagement and health behavior separately	Empirical validation (Section 5.2)

Note: Each paradigm supplies one or two stages of the loop in Figure 1; the two connections in the final row, communication infrastructure as a sensing instrument and behavioral prediction as an input to clinical risk, are made by none of the four. "Left unspecified for adherence" identifies what each paradigm would need to add to close the loop for this use case. EHR, electronic health record.

Supplementary References

24. Sutton RT, Pincock D, Baumgart DC, Sadowski DC, Fedorak RN, Kroeker KI. An overview of clinical decision support systems: benefits, risks, and strategies for success. *npj Digit Med* (2020) 3:17. doi: 10.1038/s41746-020-0221-y

30. Institute of Medicine. *Best Care at Lower Cost: The Path to Continuously Learning Health Care in America*. Washington, D.C.: National Academies Press. (2013). doi: 10.17226/13444

31. Nahum-Shani I, Smith SN, Spring BJ, Collins LM, Witkiewitz K, Tewari A, Murphy SA. Just-in-Time Adaptive Interventions (JITAIs) in Mobile Health: Key Components and Design Principles for Ongoing Health Behavior Support. *Annals of Behavioral Medicine* (2018) 52:446–462. doi: 10.1007/s12160-016-9830-8

32. Torous J, Kiang MV, Lorme J, Onnela J-P. New Tools for New Research in Psychiatry: A Scalable and Customizable Platform to Empower Data Driven Smartphone Research. *JMIR Mental Health* (2016) 3:e16. doi: 10.2196/mental.5165
